# Plant Responses and Tolerance to Salt Stress: Physiological and Molecular Interventions

**DOI:** 10.3390/ijms23094810

**Published:** 2022-04-27

**Authors:** Mirza Hasanuzzaman, Masayuki Fujita

**Affiliations:** 1 Department of Agronomy, Faculty of Agriculture, Sher-e-Bangla Agricultural University, Dhaka 1207, Bangladesh; 2 Laboratory of Plant Stress Responses, Department of Plant Science, Faculty of Agriculture, Kagawa University, Takamatsu 761-0795, Kagawa, Japan

Salinity is considered one of the most devastating environmental stresses that drastically curtails the productivity and quality of crops across the world. More than 20% of the world’s cultivable lands are dealing with the adversity of salt stress and these salt-prone areas are continuously increasing, due to both natural and anthropogenic activities [1]. However, this adversity has become much more severe in arid and semi-arid regions over the last 20 years due to the increasing demand for irrigation water requirements [2].

Salt stress is a major environmental stress that affects plant growth and development. Salt stress increases the intracellular osmotic pressure and can cause the accumulation of sodium to toxic levels [3]. Like other abiotic stresses, salt stress negatively affects plant growth and reproduction in many ways. It produces nutritional and hormonal imbalances, ion toxicity, oxidative and osmotic stress, and an increase in plant susceptibility to diseases. However, plants can be damaged or even die due to salt stress in three major ways. High salt concentration in the soil alters soil porosity and hydraulic conductivity that leads to low soil water potential; this causes water stress as a result of decreased soil water potential, eventually leading to physiological drought conditions and the destabilization of the cell membrane and protein degradation due to the toxic effects of different ions (mainly Na^+^).

Coupled with the abovementioned effects, salt stress produces a variety of physiological and metabolic changes in plants, seed germination behavior, photosynthesis, other biosynthetic process inhibition, and growth reduction. Different crops respond to salinity in different ways; glycophytes mostly show growth and total yield reduction, whereas halophytes can grow and reproduce easily under saline conditions. Therefore, at higher osmotic pressures in the root–soil interface, there is a slower impact caused by the build-up of Na^+^ and Cl^−^ in the foliage. This leads to reduced shoot growth coupled with reduced leaf expansion and the inhibition of lateral bud formation. In response to salt stress, plant cells accumulate compatible solutes and redistribute ions, which enables them to acclimate to a low soil water potential. Additionally, the endogenous abscisic acid (ABA) content increases, followed by changes in principle genetic expression in salt stress conditions. Moreover, increased levels of ions, such as Na^+^ and Cl^−^, trigger ionic toxicity in plants due to the disruption in ion homeostasis and the unavailability of essential nutrients, which are essential for plant growth and metabolism [4]. The combined effect of osmotic stress and ion toxicity is responsible for the generation of secondary stresses, which could have impaired the germination, growth, and development of the plants [5]. Salt-induced water deficit conditions declined stomatal conductance, thus reducing photosynthetic activities of the plants and accelerating the accumulation of reactive oxygen species (ROS). These are the oxygen radicals and their derivatives, such as hydrogen peroxide, H_2_O_2_; singlet oxygen, ^1^O_2_; superoxide radicals, O_2_^•−^; and hydroxyl radicals, OH^•^, which are highly reactive and usually toxic. They are capable of disrupting different cellular components, such as proteins, lipids, and nucleic acids and destructing the structural integrity of the plants [6].

On contrary, NaCl may also be used as an elicitor. Hawrylak-Nowak et al. [7] applied an abiotic elicitor, i.e., NaCl, to enhance the biosynthesis and accumulation of phenolic secondary metabolites in *Melissa officinalis* L. Plants were subjected to salt stress treatment by the application of NaCl solutions (50 or 100 mM) to the pots. Generally, the NaCl treatments were found to inhibit the growth of plants, simultaneously enhancing the accumulation of phenolic compounds (total phenolics, soluble flavonols, anthocyanins, phenolic acids), especially at 100 mM NaCl. However, the salt stress did not disturb the accumulation of photosynthetic pigments and the proper functioning of the PS II photosystem [7].

In nature, plants usually produce secondary metabolites as a defense mechanism against environmental stresses. Different stresses determine the chemical diversity of plant-specialized metabolism products. In this study, we applied an abiotic elicitor, i.e., NaCl, to enhance the biosynthesis and accumulation of phenolic secondary metabolites in *Melissa officinalis* L. Plants were subjected to salt stress treatment by application of NaCl solutions (0, 50, or 100 mM) to the pots. Therefore, the proposed method of elicitation represents a convenient alternative to cell suspension or hydroponic techniques as it is easier and cheaper with a simple application in lemon balm pot cultivation. The improvement of lemon balm quality by NaCl elicitation can potentially increase the level of health-promoting phytochemicals and the bioactivity of low-processed herbal products.

Plants are sessile and thus have to develop suitable mechanisms to adapt to high-salt environments. Thus, in response to salt stress signals, plants adapt via various mechanisms, including regulating ion homeostasis, activating the osmotic stress pathway, mediating plant hormone signaling, and regulating cytoskeleton dynamics and cell wall composition. Unraveling the mechanisms underlying these physiological and biochemical responses to salt stress could provide valuable strategies to improve agricultural crop yields. Plants, therefore, alter physiologically to deal with this situation. In species exposed to salt stress, common responses include an increase in osmotic adjustment, changes in cell wall elasticity, and an increase in the percentage of apoplastic water, which minimizes saline effects by maintaining foliar turgidity. Several compounds that work in the osmotic regulation of plants are well known, e.g., carbohydrates (sucrose, sorbitol, mannitol, glycerol, pinitol), nitrogen molecules (proteins, betaine, glutamate, aspartate, glycine, proline, choline, 4-gamma aminobutyric acid), and organic acids (malate and oxalate). Proline (Pro) and glycine betaine (GB) are the most essential and efficient compatible solutes among the organic osmolytes which minimize the salt-stress effects and improve plant growth.

Plant metabolic plasticity is also a vital factor in regulating salt stress response in plants. Nosek et al. [8] analyzed the photosynthetic metabolism of the ice plant (*Mesembryanthemum crystallinum* L.) during a 72 h response period following salinity stress removal and found that the presence of salinity stress is required not only for the induction of stress-dependent crassulacean acid metabolism (CAM) photosynthesis but also for maintaining its functioning. The rapid shutdown of the energy-demanding functional CAM seems to be one small component of the flexibility features. As we showed here, the metabolic flexibility of the common ice plant includes rapid and far-reaching changes [8].

It is essential to carry out the necessary research on salt stress and soil pollution mitigation to increase agricultural productivity and feed the world’s growing population. As global hunger and salinity stress intensify, there is a rise in interest in ecologically sustainable solutions for salt tolerance. Apart from using freshwater for irrigation there are many other alternative approaches for developing salt-tolerant crops. Developing halophytes as alternative crops, using interspecific hybridization to improve current crop tolerance, utilizing the wide range of varieties of existing crops, the introduction of variation within existing crops through genetic approaches, or improving salt-tolerant varieties.

Plant growth-promoting bacteria, phytohormones, and organic acids can also be used to promote salt tolerance in plants. Moreover, the improvement and/or alteration of some agricultural management practices will also play a vital role in the mitigation of salt stress in the first place. For example, conventional crop and pasture breeding for salt tolerance, soil reclamation, various management practices based on reducing the salt zone for seed germination and seedling establishment, pre-sowing irrigation with good quality water, appropriate use of ridges or beds for planting, and general management practices (mulching, incorporation of organic matter) will reduce the impact of soil salinity on crop performance.

Surowka et al. [9] reported that salt-exposed α-TC accumulating plants were more flexible in regulating chlorophyll, carotenoid, and polysaccharide levels than TC deficient mutants, while the plants overaccumulating γ-TC had the lowest levels of these compounds. They found that salt-stressed TC-deficient mutants and *tmt* transgenic line exhibited greater proline levels than WT plants, lower chlorogenic acid levels, and lower activity of catalase and peroxidases. Plants that accumulate α-TC produced more methylated proline and glycine betaines and showed greater activity of superoxide dismutase than γ-TC deficient plants [9].

Different plant hormones and genes are also associated with the signaling and antioxidant defense system to protect plants when they are exposed to salt stress. Salt-induced ROS overgeneration is one of the major reasons for hampering the morpho-physiological and biochemical activities of plants which can be largely restored by enhancing the antioxidant defense system that detoxifies ROS [6].

Reactive oxygen species signaling is crucial in modulating stress responses in plants, and NADPH oxidases (NOXs) are an important component of signal transduction under salt stress [10]. Pilarska et al. [10] showed that salt-induced expression patterns of two NOX genes, *RBOHD* and *RBOHF*, varied between the halophyte and the glycophyte. The expression of these genes in *E. salsugineum* leaves was induced by abscisic acid (ABA) and ethephon spraying indicates that in the halophyte *E. salsugineum*, the maintenance of the basal activity of NOXs in leaves plays a role during acclimation responses to salt stress.

Combining genomics and transcriptomics is a new approach that deals with the deep understanding and knowledge of the molecular response of a plant to salinity. Modification of genetic expression in salt stress comprises a variety of ways that plants use to induce or suppress the transcription of genes.

Quertani et al. [11] performed transcriptomic analysis of salt-stress-responsive genes in barley and found that, in leaves, the expression of 3585 genes was upregulated and 5586 were downregulated, while in roots the expression of 13,200 genes was upregulated and 10,575 were downregulated. They also found that response to salt stress is mainly achieved through sensing and signaling pathways, strong transcriptional reprogramming, hormonal regulation, osmoregulation, ion homeostasis and increased ROS scavenging. A number of candidate genes involved in hormone and kinase signaling pathways, as well as several transcription factor families and transporters, were identified. This study provides valuable information on early salt-stress-responsive genes in the roots and leaves of barley and identifies several important players in salt tolerance [11].

Proteomic and metabolomic research provides an excellent tool for examining plant adaptation to salinity. Hence, these are frequently utilized to identify the molecular processes of plant response to salt stress.

In mulberries, Gan et al. [12] performed a comparative proteomic analysis of salt tolerant and sensitive varieties and revealed that the phenylpropanoid biosynthesis may play an important role in the salt tolerance of the mulberry. They also clarified the molecular mechanism of mulberry salt tolerance, which is of great significance for the selection of excellent candidate genes for saline–alkali soil management and mulberry stress resistance genetic engineering [12].

He et al. [13] identified *bolTLP1*, a thaumatin-like protein gene that confers tolerance to salt stress in broccoli (*Brassica oleracea* L. var. *Italica*) because *bolTLP1* may function by regulating phytohormone (ABA, ethylene, and auxin)-mediated signaling pathways, hydrolase and oxidoreductase activity, sulfur compound synthesis, and the differential expression of histone variants [13].

However, more information as well as research on genetic engineering, transcriptomics, proteomics, and metabolomics research, as well as their combined responses, is required to determine the salt-tolerance mechanism of plants. Identification of rice salt-tolerance genes and their molecular mechanisms could help breeders genetically improve salt tolerance [14]. Genome-wide identification and functional characterization of the cation proton antiporter (CPA) family were also found to be effective in providing insight into salt tolerance [15]. Wang et al. [15] identified 60 CPA candidate genes of radish on the whole genome level and concluded that these results would be useful to understand the complexity of the RsCPA gene family and could provide a valuable resource to explore the potential functions of RsCPA genes in radish. Similarly, Chen et al. [16] performed genome-wide analysis of the apple CBL family and revealed that Mdcbl10.1 functions positively in modulating apple salt tolerance. It also provided valuable insights for future research examining the function and mechanism of CBL proteins in regulating apple salt tolerance [16].

The study of Shao et al. [17] provided fundamental information on the involvement of the wheat *14-3-3* family in salt stress. They identified a total of 17 potential *14-3-3* gene family members that were identified from the Chinese Spring whole-genome sequencing database. Importantly, most *14-3-3* members in wheat exhibited significantly downregulated expression in response to alkaline stress [17].

Considering the importance of the physiological functions of melatonin, Tan et al. [18] studied the expression of the melatonin-related gene *HIOMT* in apple plants induced by salinity. They found that compared with the wild type, transgenic lines indicated higher melatonin levels and showed reduced salt damage symptoms, lower relative electrolyte leakage, and less total chlorophyll loss from leaves under salt stress. Further, transgenic lines showed a lower amount of ROS due to the enhanced activity of antioxidant enzymes, downregulated the expression of the abscisic acid synthesis gene (*MdNCED3*), accordingly reducing the accumulation of abscisic acid under salt stress [18].

Zhang et al. [19] characterized the *PsnNAC036* gene and found that the overexpression of *PsnNAC036* stimulated plant growth and enhanced salinity and heat tolerance in *Populus simonii × P. nigra*.

Next generation gene sequencing opens the avenue to revealing salt tolerance mechanisms more precisely. Min et al. [20] reported that haplotype analysis of *BADH1* by next-generation sequencing reveals an association with salt tolerance in rice during domestication. Despite the unclear association between *BADH1* and salt stress, these findings can be useful for future research development related to its gene expression [20].

Katja et al. [21] detected jacalin-related lectin HvHorcH protein in root extracellular fluid, suggesting that the revealed expression of HvHorcH is involved in the adaptation of plants to salinity.

Yu et al. [22] found that the C2H2-type zinc-finger protein from *Millettia pinnata*, MpZFP1, is a positive regulator of plant responses to salt stress due to its activation of gene expression and efficient scavenging of ROS and enhances salt tolerance in transgenic *Arabidopsis*. The heterologous expression of *MpZFP1* in Arabidopsis increased the seeds’ germination rate, seedling survival rate, and biomass accumulation under salt stress [22].

The plant cytoskeleton is associated with plant salt stress responses. Therefore, the molecular mechanism underlying microtubule functions in plant salt stress response is important. Chun et al. [23] found that microtubule dynamics play crucial roles in plant adaptation and tolerance to salt stress. The modulation of microtubule-related gene expression can be an effective strategy for developing salt-tolerant crops [23].

Overall, the 19 contributions in this Special Issue “Plant Responses and Tolerance to Salt Stress: Physiological and Molecular Interventions” discuss the various aspects of salt stress responses in plants. It also discusses various mechanisms and approaches to conferring salt tolerance on plants. These types of research studies provide further directions in the development of crop plants for the saline environment in the era of climate change.

## Data Availability

Not applicable.
